# At the Intersection of Pain and Sleep: a Roadmap for Preclinical Pain Research

**DOI:** 10.3389/fpain.2025.1609524

**Published:** 2025-10-20

**Authors:** Clare M. Diester, William Joo

**Affiliations:** Biozentrum, University of Basel, Basel, Switzerland

**Keywords:** sleep, pain, pain and sleep comorbidity, pain and sleep interference, neural circuitry, translational research

## Abstract

The complex relationship between pain and sleep has received increasing attention for its therapeutic potential. Over half of chronic pain patients suffer from sleep disorders, and poor sleep is a strong predictor for pain in clinical populations. Understanding the bidirectional relationship between pain and sleep is crucial for developing improved clinical treatment strategies. This review provides (1) a primer on preclinical methods used to measure sleep behaviors, (2) an overview of neural circuits at the intersection of pain and sleep, and (3) considerations for future pain and sleep investigations and treatment strategies.

## Introduction

Chronic pain affects millions of people globally and is even more prevalent than other chronic conditions such as diabetes and hypertension (1, 2). Impaired sleep and fatigue are primary complaints for most chronic pain patients (3–5). Indeed, patients suffering from chronic pain exhibit significant sleep disturbances such as fragmented sleep, non-restorative sleep, disrupted sleep architecture, difficulties initiating sleep, pain-related arousals from sleep, and abnormally shallow sleep (2, 3, 6). Within chronic pain patients, the degree of sleep disturbance is associated with pain severity (7–10). Conversely, application of acute noxious stimuli to healthy subjects has been shown to be equally disruptive across all sleep stages in controlled lab environments (11–13).

A large portion of the general population also suffers from sleep disorders, with insomnia being the most common (∼10%–15% of the general population) (14). Roughly 40% of insomnia patients also report chronic pain (6). Short sleep duration or disturbed sleep can increase spontaneous pain, enhance pain sensitivity, and amplify pain-elicited neuronal responses, in part by disrupting pain modulatory systems (2, 6, 8, 9, 11, 15, 16). Furthermore, sleep disturbances or sleep disorders are associated with chronic postsurgical pain or exacerbation of existing pain, while patients with poor sleep exhibit increased risk of developing chronic pain (6, 17, 18).

While sleep and pain have been extensively studied within their respective fields, the dynamic interplay between pain and sleep remains poorly understood. This review is targeted to preclinical pain researchers interested in adding sleep as a primary outcome variable to their studies. Below, we summarize approaches to study sleep, brain regions that may regulate pain and sleep, and key considerations for future experimental design. For more comprehensive discussions on the state of sleep research or the neurochemical mechanisms, inflammatory interactions, and pharmacological modulation of pain and sleep, we refer readers to other excellent reviews (7, 9, 19–23).

## Methods to measure sleep

Sleep is a highly conserved biological phenomenon, with well-defined methods to evaluate different facets of sleep-wake behavior (19, 20, 24–27). Methods range in sensitivity, reliability of sleep-related interpretations, and ease of implementation alongside existing common pain-related behavioral measures. Below, we discuss experimental approaches to measure sleep-wake behavior, with comments on integration into pain-related behavioral measures.

### Dependent variable

**Figure 1:**
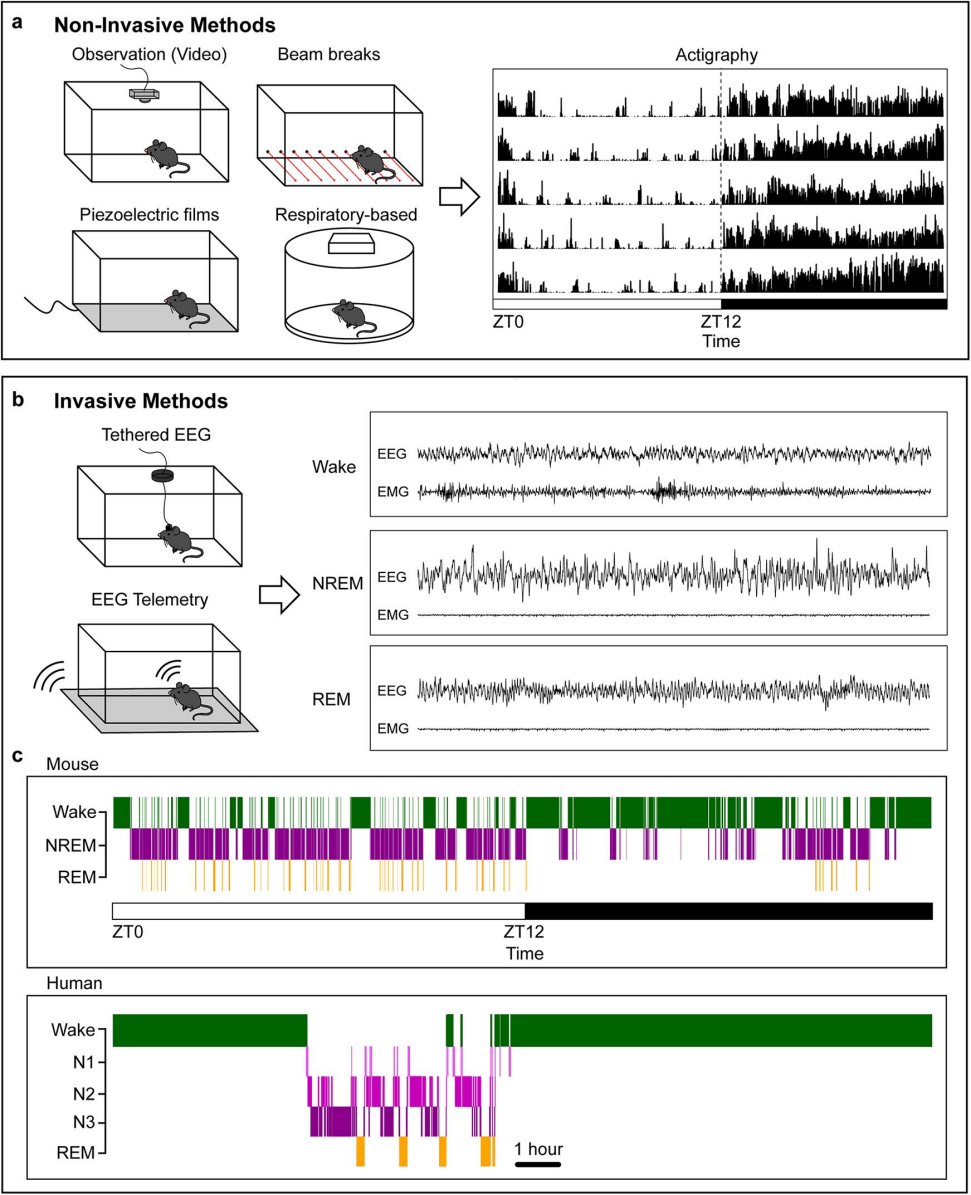
**Common methods for measuring sleep.** **(a)**, Example non-invasive methods for classifying sleep behavior in rodents and example 5-day actigraphy data. Rows represent consecutive days; the dashed line indicates the transition from light to dark phase. The white bar indicates light phase starting at zeitgeber time (ZT) 0, the black bar indicates dark phase starting at ZT12. **(b)**, Example invasive methods for evaluating sleep/wake architecture and sample mouse EEG and EMG recordings during the three vigilance states: Wake, NREM, and REM. **(c)**, Sample hypnogram data for a mouse (top) and human (bottom). Human data are from the Sleep-EDF database (196, 197). Mice sleep more during the light phase with frequent arousals and often take a “siesta” at the end of their active dark phase. Humans have three NREM phases increasing in sleep depth. Sleep and wake behavior is highly consolidated, with rare arousals during the night in healthy individuals.

The first class of behavioral measures for preclinical sleep-related endpoints are non-invasive observational approaches. These can monitor locomotion via standard behavioral methods such as video tracking, beam breaks, or wheel running, along with techniques such as piezoelectric films that can capture additional features such as respiratory rate and heart rate, as depicted in Figure 1 (28–32). Advancements in markerless pose estimation tools such as DeepLabCut (DLC) and Social LEAP (SLEAP) can classify additional behaviors such as sleep preparatory behaviors and multi-animal social sleep behavior (33–36). Newer devices merge the non-invasive nature of home-cage monitoring with more refined sleep/wake analyses, such as sleep/wake phenotyping based on respiratory patterns (37). However, most of these methods cannot classify Rapid-Eye Movement (REM) sleep or other fine-grained properties of sleep (24, 37). Additionally, methods such as voluntary wheel running can evaluate circadian rhythms but can also change baseline sleep-wake behavior in the absence of other perturbations (38). Overall, non-invasive methods are attractive because they do not require surgery and can easily be integrated with existing behavioral paradigms. However, these techniques primarily measure physical activity-based correlates of sleep and wake, especially for locomotion-based assays. Given that rodents frequently enter low-mobility wake states, true “sleep” should not be assumed based on locomotor quiescence without additional measures and/or validation with EEG data. Accordingly, these measures have been recommended for high-throughput screens with secondary methods for further evaluation of sleep architecture (30, 31).

The second class of sleep measures requires surgical procedures for classical evaluation of brain electrical activity. In mammals, tethered recording of electroencephalography (EEG) and electromyography (EMG) data has been the gold-standard for sleep state classification in preclinical research (19, 20, 39, 40). Surgical procedures secure EEG electrodes in the skull over key cortical regions, as well as EMG electrodes in the neck extensor muscle to capture postural tone (39, 40). These components are joined to an external implant on the head with a flexible cable (with a rotating commutator if desired) that relays EEG and EMG signal.

Wireless telemetry-based methods offer a non-tethered alternative in which EEG and EMG electrodes connect to a surgically implanted transmitter, which links to a receiver under or near the animal cage. Telemetry allows animals to move more freely during experiments but can be more complex to build and often relies on expensive consumables. Battery life is another consideration when designing longitudinal experiments (41). Additionally, care should be taken to ensure that implanted transmitter devices do not impact baseline behaviors, as larger devices can increase surgery recovery time and chronically disrupt home cage behaviors (42). Recent efforts have focused on reducing implant size and incorporating multiple biopotential leads to combine EEG with additional physiological measures. Both tethered and telemetry-based approaches can be seamlessly integrated with video monitoring and implant-based neuroscience methods such as optogenetics, fiber photometry, extracellular electrophysiology, functional ultrasound imaging, multimodal fibers, and *in vivo* microscopy (43–47). As with any surgical manipulation, animals are inherently subjected to surgical pain requiring pharmacological treatment. Accordingly, researchers should ensure full recovery and stable post-surgical sleep/wake behavior before conducting pain-related experiments. While both involve basic surgical training and additional equipment such as amplifiers or receivers, these techniques can connect external behavioral readouts with concordant brain-state activity to better understand whole-organism behavioral states.

### Sleep stage classification

Standardized methods for analyzing sleep and wake data do not currently exist within the preclinical sleep field. Instead, many labs use custom software and analysis parameters tailored to specific questions. Historically, EEG and EMG data have been visually scored by trained experts, which can produce variability between analysts and is extremely time consuming, often restricting the length of the experimental time window (24, 39). Continuous advances in automated sleep classification based on EEG and EMG data now allows easier analysis of long time periods (days and weeks), removes between-analyst sleep scoring variability, and reduces data processing time (24). However, these automated classifiers are not 100% accurate and are often paired with partial manual scoring (24). Regardless of scoring method, EEG and EMG data are typically divided into three main states: Wake, Non-REM sleep (NREM), and Rapid-Eye Movement sleep (REM), with further details described below.

### Baseline circadian vs. sleep disruption

First proposed by Borbély in 1982, the two-process model proposes that sleep is regulated by two separate but interacting systems: A circadian system (Process C) that aligns sleep/wake behaviors with day/night cycles, and a homeostatic system (Process S) that adjusts sleep depth and amount according to prior sleep/wake history (48). In general, noxious stimuli can be evaluated during baseline circadian behavior or in the context of homeostatic sleep disruption. Importantly, both noxious stimuli and sleep loss can be conducted acutely or chronically. Given that pain and sleep are both multifaceted phenomena that impact numerous physiological systems, their interaction introduces additional layers of complexity that require careful consideration in experimental design. Below are some considerations for designing experiments, divided into the implications of noxious stimuli applied during baseline circadian behavior or sleep disruption.

Baseline Circadian: Baseline behavior critically includes circadian regulation of biological systems, including core processes involved in sleep and pain regulation (49–52). Preclinical and clinical work both highlight the large impact of circadian rhythms on pain-related responses (51, 53). For example, Daguet and colleagues unmasked circadian rhythmicity in sensitivity to noxious heat stimuli in men, showing a sinusoidal rhythm with peak responses in the middle of the night and lowest responses in the afternoon (54). Circadian rhythmicity of acute nociception has long been demonstrated in rodents, including strain-based differences in peak pain sensitivity in mice. For instance, inbred C57BL/6 mice (one of the most commonly used strains in preclinical research) exhibit increased dark-phase sensitivity, while outbred Swiss Webster mice exhibit peak sensitivity during the light-phase (51, 55–57). Modulating circadian rhythmicity can impact pain-related behaviors, as exemplified by changes in mechanical allodynia after misaligned feeding in mice (58). Specific circadian timing of surgical procedures may influence patient outcomes. For example, aortic valve replacements and hip replacements have been associated with fewer adverse effects when surgeries were performed in the afternoon (59, 60). In rodents, some studies report enhanced wound healing following morning injuries; however, not all operations may have circadian sensitivity, as surgical timing did not affect pain outcomes in models of paw incision or tibial bone fracture (61–63). Moreover, time of day will strongly influence floor and ceiling effects given the natural circadian distribution of sleep/wake behavior. Accordingly, circadian timing should be carefully considered when assessing pain/sleep interactions.

Sleep Disruption: Preclinical and clinical studies have clearly demonstrated a bidirectional relationship between pain and sleep loss. For example, both acute and chronic sleep deprivation can alter acute pain sensitivity and exacerbate chronic pain, while poor sleep quality can predict following-day pain. In some cases, treatment of sleep disruptions can improve pain outcomes. Conversely, acute and chronic pain can alter sleep architecture, and chronic pain is a significant risk factor for developing sleep disorders (2, 6–9, 11, 15, 64–69).

Preclinical studies use numerous methods to disrupt sleep. Manual sleep deprivation methods include novel objects exposure and gentle handling (70, 71). Automated methods include a slowly rotating bar at the bottom of the cage, shaking platforms, and raised surfaces above shallow water, and may be more feasible for longer deprivation studies (70, 72). Sleep disruption methods typically aim to (a) robustly decrease sleep over the desired period, and (b) minimize stress, particularly since stress has been shown to modulate both pain and sleep (70). Corticosterone levels are a commonly used readout of stress induction (70). Sleep disruption-induced stress will likely vary by method and laboratory setup, as exemplified by a recent study that found sleep deprivation with an automated sweeping bar significantly increased corticosterone levels while gentle handling did not (73).

The duration of the sleep manipulation can drastically impact pain-related processes. For example, 9 h and 12 h sleep disruption produced pain-related behaviors in mice, but 6 h sleep disruption only produced allodynia when repeated for five consecutive days (74). Pain-related response magnitude may also change over the course of chronic sleep restriction. For instance, during a 26-day chronic 6hr sleep restriction, mechanical allodynia increased up to 12 days, where it remained stable until day 26 (75). Finally, timing for behavioral testing around sleep manipulations is important and will depend on the research question. Clear time windows for evaluation of sleep disruption-induced pain should be determined, especially for cases where the hypersensitivity naturally resolves (74). Effects of perioperative sleep disruption on post-surgical pain can also be differentially evaluated depending on whether the sleep disruption occurs before, after, or surrounding a surgical procedure (76–81).

## Overview of general sleep architecture

### States

EEG and EMG data are generally classified into three main states: (1) Wake, with high-frequency, low amplitude desynchronized EEG activity paired with EMG reflecting active changes in muscle tone; (2) Non-REM sleep (NREM), with synchronized low-frequency, high-amplitude EEG activity; and (3) Rapid-Eye Movement sleep (REM, also called paradoxical sleep), with desynchronized high-frequency, low amplitude activity similar to that of wake, and reduced EMG signal reflecting muscle atonia (19, 20, 32). In rats, NREM can be divided into two stages reflecting higher or lower sleep depth (19). For mice and rats, wake can also be further divided into active or inactive wake based on EEG and EMG properties or secondary measures, such as video observation.

In humans, NREM sleep is divided into three stages based on increasing sleep depth: N1-N3 (82). Sleep and wake in humans are highly consolidated, with very few night awakenings and continuous wake during the day, as demonstrated in Figure 1. In contrast to humans, mice sleep more during the light phase and have more fragmented NREM and REM sleep cycles with frequent awakenings during their resting phase. Mice also have less consolidated sleep than humans and sleep during their active phase. Despite these differences, EEG features and brain regions regulating sleep and wake states show remarkable conservation between the two species (19, 20).

### Spectra

EEG oscillations span a wide range of frequencies that are often divided into behaviorally meaningful ranges, such as delta waves (∼0.5–4.5 Hz), theta oscillations (∼5–10 Hz), and gamma oscillations (∼30–150 Hz). The power distribution across these frequency ranges is highly characteristic of different vigilance states. For example, a hallmark of NREM sleep are high-amplitude slow delta oscillations (which is why NREM is also called Slow Wave Sleep), while Wake and REM EEG are predominantly comprised of desynchronized low-amplitude, high-frequency activity. EEG characteristics alongside EMG data result in robust and highly reliable classification of sleep and wake states (19, 20). Preclinical and clinical evidence indicate that chronic pain can alter these frequency bands; however, these effects may vary by pain indication and manifest as global or regionally specific spectral changes (83–88). Researchers are currently working to determine the utility of these EEG frequency bands for use as a diagnostic, monitoring tool, or treatment for chronic pain patients.

### Additional features

Depending on the research question, additional EEG features may add important insights to pain and sleep interactions. For example, microarousals (3- to 15-second-long wake intrusions into NREM sleep) have recently been described as disrupting restorative and plasticity-promoting sleep (89). Preclinical neuropathic pain—but not inflammatory or chemical pain—produced microarousals and higher sensory arousal during NREM sleep without changing total sleep time, offering a possible biomarker for spontaneous neuropathic pain in mice (69, 89, 90). Sleep spindles (∼10–16 Hz), which fluctuate in 0.5–2.0 s oscillations during NREM sleep, are one of the most heritable components of EEG signatures, and likely reflect properties of the underlying thalamocortical circuits (19, 91, 92). Recent preclinical work has demonstrated that both acute inflammatory pain in mice and chronic inflammatory pain in rats reduces sleep spindle density, and alleviating pain-related behaviors also restored sleep spindle activity, suggesting sleep spindles as a possible marker for acute or chronic pain (93, 94).

## Neuronal circuitry implicated within the intersection of pain and sleep

Extensive work has independently characterized neural circuits of acute and chronic pain and the neural mechanisms regulating sleep and wake (19, 20, 95–105). As other reviews have described the neuroanatomy of either pain or sleep (19, 20, 95–105), this section focuses on neuronal mechanisms directly linking pain and sleep regulation. Relevant anatomy is categorized by the following pain and sleep interactions: (1) Acute noxious stimuli during baseline sleep/wake, (2) Acute noxious stimuli with acute sleep disruption (3) Acute noxious stimuli with chronic sleep disruption, (4) Chronic pain during baseline sleep/wake, (5) Chronic pain with acute sleep disruption, and (6) Chronic pain with chronic sleep disruption.

Acute noxious stimuli during baseline sleep/wake: Acute noxious stimuli applied during wake engage the widely studied ascending and descending pain circuitry, which has been comprehensively reviewed elsewhere (51, 95–98, 104, 105). Application of acute noxious stimuli during sleep in both humans and rodents show consistent arousal-promoting activity in sensory regions. Noxious stimulus-induced arousal can be predicted by intracortical functional connectivity, and locus coeruleus noradrenergic activity may be an important mediator for such stimulus-induced arousal (106–109). Recent work has shown that both noxious and innocuous stimulus-induced activity sequentially activate the somatosensory cortex (S1) and anterior cingulate cortex (ACC) independent of behavioral response (110). Interestingly, this sequential somatosensory processing is conserved during uninterrupted sleep bouts. This suggests that somatosensory processing is preserved during sleep even in the absence of visible behavioral responses.

**Figure 2:**
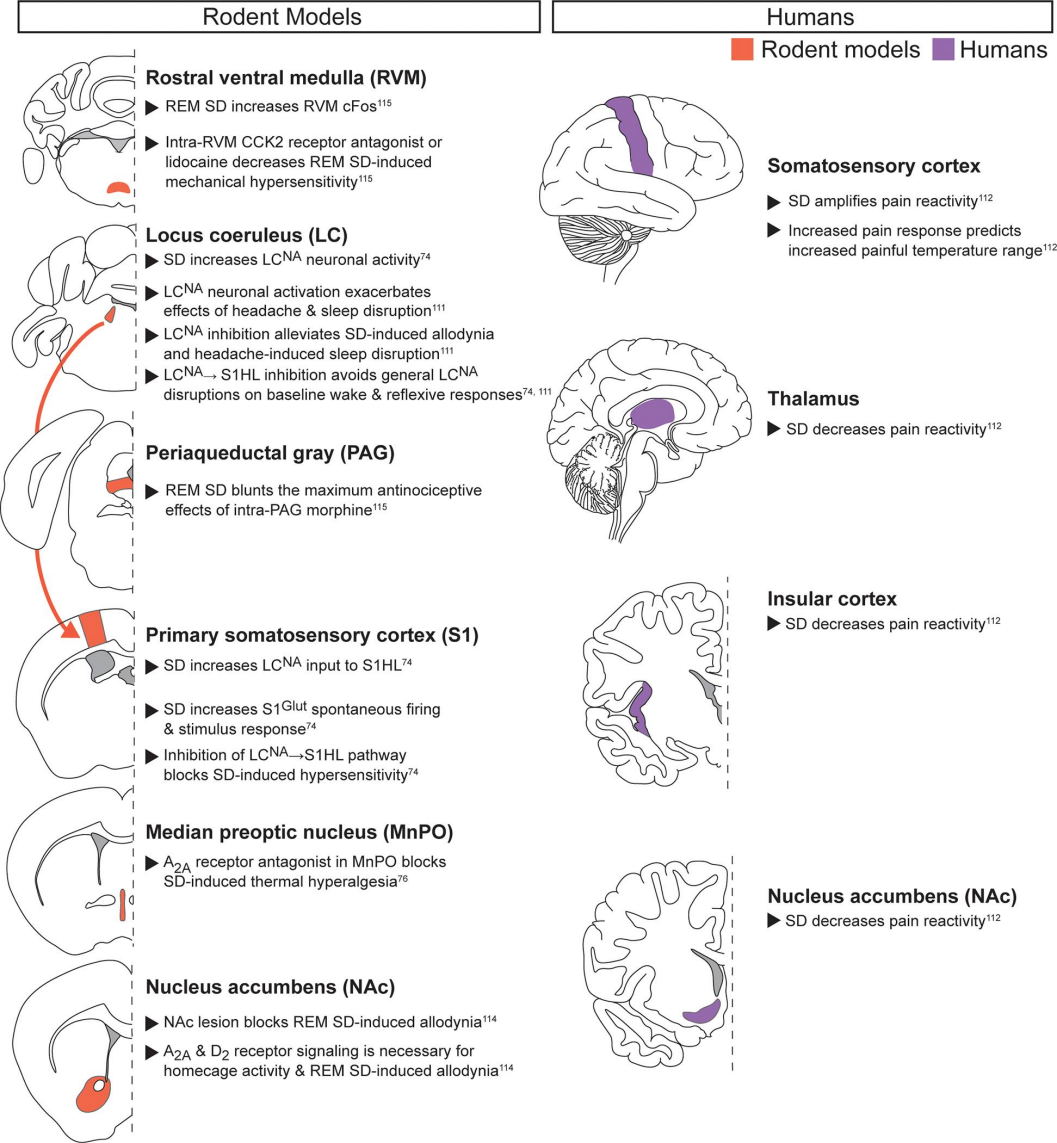
**Acute noxious stimuli with acute sleep disruption.** Neuroanatomical regions implicated in regulating the interaction between pain and sleep in rodents (left column) and humans (right column) from studies pairing acute noxious stimuli with acute sleep disruption. Detailed descriptions are in the corresponding text section, and references are in superscript. SD, sleep disruption; S1HL, hindlimb primary somatosensory cortex; Glut, glutamatergic.

Acute noxious stimuli with acute sleep disruption (Figure 2): Preclinical and clinical evidence both indicate that acute sleep disruption alters pain-related neuronal activity in the S1 (8, 9, 65, 74, 111, 112). In humans, acute sleep disruption amplified S1 pain responses, and the degree of amplification predicted increased painful temperature range across individual patients (112). Recent preclinical work has directly linked increased S1 activity after acute sleep deprivation to increased activity in the locus coeruleus (LC), a region that regulates wake and REM via noradrenergic signaling (74). Specifically, noradrenergic neurons in the LC (LC^NA^) project to glutamatergic neurons in hindlimb S1 (S1HL^Glut^). After a 9hr acute sleep deprivation, LC^NA^ neurons release more noradrenaline (NA) in the S1HL, leading to elevated S1HL^Glut^ activity. Selective activation and inhibition demonstrated this LC^NA^ → S1HL pathway to be both sufficient and required for acute sleep deprivation-induced hypersensitivity and allodynia. A separate study evaluated the interactions between 24 h sleep deprivation and nitroglycerin (NTG)-induced migraine-like headache and found that LC^NA^ neuronal activation exacerbated sleep deprivation-induced amplification of acute headache behaviors while inhibition could alleviate them (111). However, LC^NA^ inhibition may decrease reflexive pain-related behaviors due to general suppression of arousal. Indeed, manipulation of all LC^NA^ neurons altered baseline sleep, with inhibition also producing general depression of pain-related reflexive behaviors in the absence of noxious stimulus or sleep deprivation. In contrast, selectively manipulating LC^NA^ → S1HL projections in rested animals did not disrupt baseline sleep or pain-related behaviors (74). This suggests targeting specific circuits rather than all LC^NA^ neurons may be key to alleviating symptoms without disrupting general behavior. Taken together, these data suggest the LC plays a pivotal role in multiple pain and sleep modalities through its arousal-promoting functions (107, 113).

Preclinical and clinical evidence also suggest that the nucleus accumbens (NAc) regulates the interaction between acute noxious stimuli and acute sleep disruption. In humans, acute sleep disruption blunts acute pain reactivity in the NAc, along with the thalamus and insular cortex (112). In rodents, acute REM sleep deprivation in rats produces allodynia, an effect potentially mediated by adenosine and dopamine signaling (114). Specifically, D_2_ receptor agonists, adenosine A_2A_ receptor antagonists, and caffeine prevented REM sleep deprivation-induced allodynia. Adenosine signaling in the MnPO also mediates pain reactivity, as adenosine A_2A_ receptor antagonist microinjection prevents both sleep disruption-induced hyperalgesia and exacerbation of postoperative hypersensitivity (see chronic pain with acute sleep deprivation section) (76). This provides further evidence that adenosine A_2A_ signaling mediates sleep deprivation-induced pain-related behaviors in response to multiple sleep disruption methods.

REM sleep deprivation also produced changes in brainstem activity, including increased cFos expression in the rostral ventral medulla (RVM), an important region for descending pain modulation (115). Microinjection of a cholecystokinin (CCK)-2 receptor antagonist or lidocaine into the RVM decreased REM sleep deprivation-induced increases in mechanical hypersensitivity, with no effect on control rats. In contrast, CCK-2 receptor agonist injected into the RVM increased hypersensitivity only in control animals. The periaqueductal gray (PAG), another region critical for descending pain modulation, is also modified following REM-specific sleep deprivation. Specifically, REM sleep deprivation decreased the maximum antinociceptive effect of intra-PAG morphine injection on mechanical hypersensitivity. Taken together, these data suggest REM-specific acute sleep disruption increases acute pain-related behaviors by disrupting descending pain modulation, impacting general hypersensitivity, and decreasing the analgesic effectiveness of morphine.

**Figure 3:**
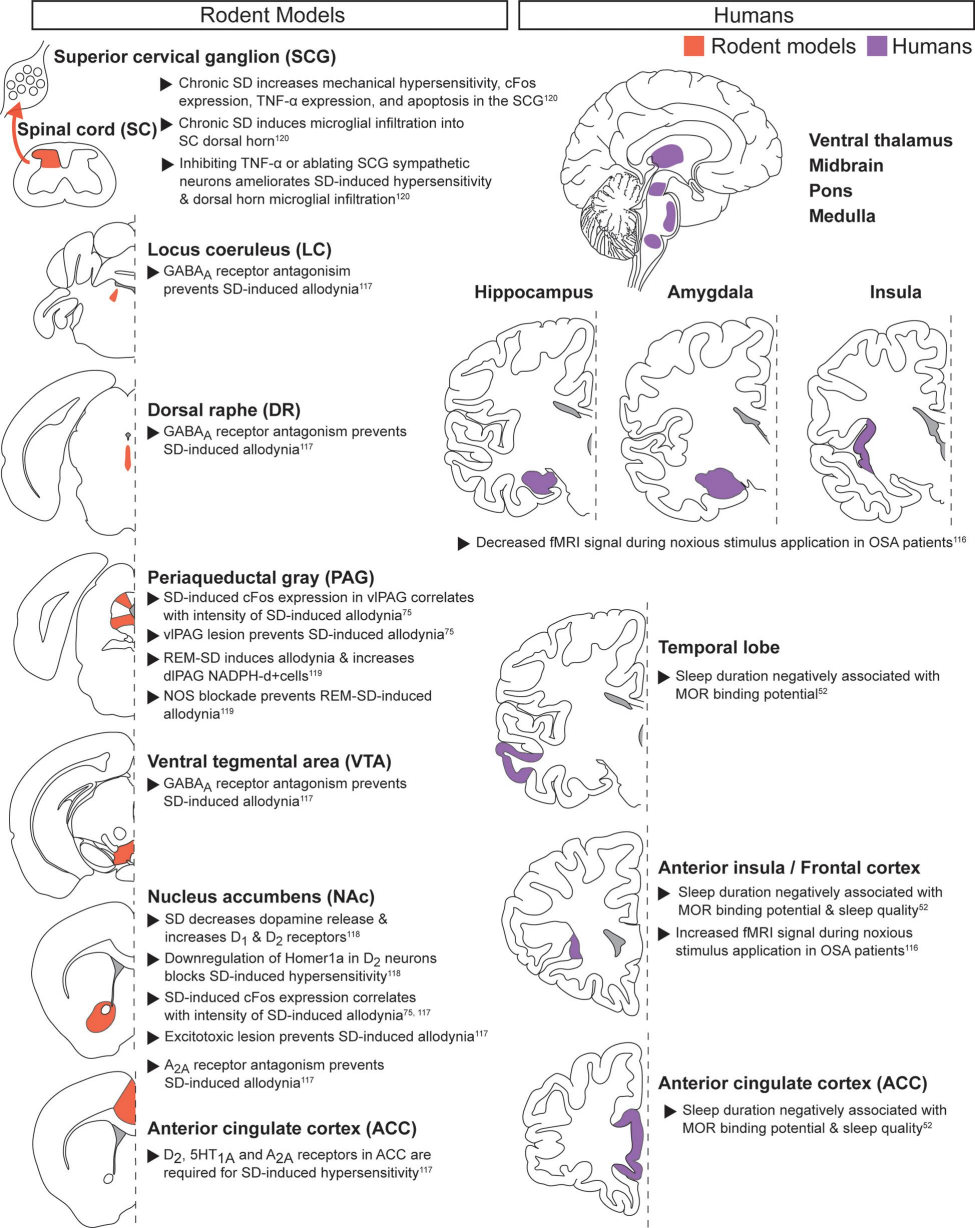
**Acute noxious stimuli with chronic sleep disruption.** Neuroanatomical regions implicated in regulating the interaction between pain and sleep in rodents (left column) and humans (right column) from studies pairing acute noxious stimuli with chronic sleep disruption. Detailed descriptions are in the corresponding text section, and references are in superscript. SD, sleep disruption; vlPAG, ventrolateral PAG; REM-SD, REM-specific sleep disruption; dlPAG, dorsolateral PAG; NOS, nitric oxide synthase; OSA, obstructive sleep apnea; MOR, mu opioid receptor.

Acute noxious stimuli with chronic sleep disruption (Figure 3): Multiple studies have examined how chronic sleep disruption alters responses to acute noxious stimuli (52, 75, 116–120). Patients with chronic sleep disorders such as obstructive sleep apnea (OSA) exhibit increased cortical fMRI signal duration during cold pressor noxious stimulus application (116). In contrast, regions including the hippocampus, amygdala, insula, ventral thalamus, midbrain, pons, and medulla showed decreased fMRI signal in OSA patients compared to controls, suggesting dynamic global changes to how OSA patients perceive acute thermal noxious stimuli.

The opioid system may contribute to the cortical changes observed in patients with chronic sleep disruption. Individual patient sleep duration is negatively associated with mu-opioid receptor binding potential in cortical regions such as the frontal lobes and anterior cingulate cortex (ACC), offering a possible mechanism for the decreased opioid efficacy observed in sleep deprived patients (52). Preclinical pharmacological work provides further evidence that ACC activity partially mediates the pronociceptive effects of chronic sleep deprivation in male rats (117). More specifically, ACC dopaminergic D_2_ receptor, serotonergic 1_A_ receptor, and adenosine A_2A_ receptor activity were each required for sleep deprivation-induced hypersensitivity.

Recent work has identified the NAc, a key region for dopamine signaling, as an important mediator in chronic sleep deprivation-induced hypersensitivity (75, 117, 118). In male rats, the immediate early gene cFos increased in the NAc after chronic sleep deprivation and returned to baseline levels during rebound sleep. cFos expression correlated with the intensity of the pronociceptive effect, and excitotoxic lesioning of the NAc prevented chronic sleep deprivation-induced hypersensitivity (75, 117). A preclinical study using male mice extended this work by demonstrating decreased dopamine release and increased D_1_ and D_2_ receptor expression in the NAc following chronic sleep deprivation (118). Post-sleep deprivation hypersensitivity may require upregulation of AMPA receptors by the immediate early gene Homer1a, as downregulating Homer1a in NAc blocked chronic sleep deprivation-induced hypersensitivity. Adenosine, a key neuromodulator of sleep and wake, may also mediate chronic sleep deprivation-induced hypersensitivity, as blocking adenosine A_2A_ receptor signaling in the NAc prevented increased reflexive-based hypersensitivity (117). Increased GABA signaling may also contribute, as sleep-restriction-induced hypersensitivity required GABA_A_ receptor signaling in the ventral tegmental area (VTA), dorsal raphe nucleus (DRN), and locus coeruleus (LC) (117). Together, these data suggest that dopamine neurons in the NAc can play a critical role in chronic sleep disruption-induced hypersensitivity through Homer1a- and adenosine A_2A_-dependent mechanisms, while inhibitory neurotransmission may exhibit distributed changes.

The PAG, a critical component of pain modulation, has also been linked to increased pain-related responses following chronic sleep deprivation. The ventrolateral portion of the PAG (vlPAG) showed increased cFos expression after chronic sleep deprivation, as in the NAc. cFos induction correlated with the intensity of deprivation-induced allodynia, and excitotoxic lesion of the vlPAG prevented increased withdrawal reflexes (75). Chronic REM sleep restriction increased nitric oxide in the dorsolateral PAG (dlPAG), and inhibition of nitric oxide synthase (NOS) prevented the chronic REM sleep restriction-induced allodynia (119). Increased nitric oxide in the PAG may thus promote chronic sleep deprivation-induced hypersensitivity. However, additional studies must evaluate other acute noxious stimuli and pain-related behaviors, along with sleep disruptions beyond REM restriction.

In addition to central brain mechanisms, spinal cord signaling can also mediate chronic sleep deprivation-induced hypersensitivity (120). Chronic sleep restriction that produced hypersensitivity in male mice increased microglial infiltration into the spinal cord dorsal horn and increased both apoptosis and TNF-α expression in superior cervical ganglion (SCG) sympathetic neurons. Blocking TNF-α receptors prevented these effects as well as allodynia, suggesting sympathetic SCG neurons promote hypersensitivity following chronic sleep restriction (120). These results highlight how peripheral inflammatory signaling may contribute to supraspinal hyperactivity after chronic sleep deprivation (7, 9, 121, 122).

**Figure 4:**
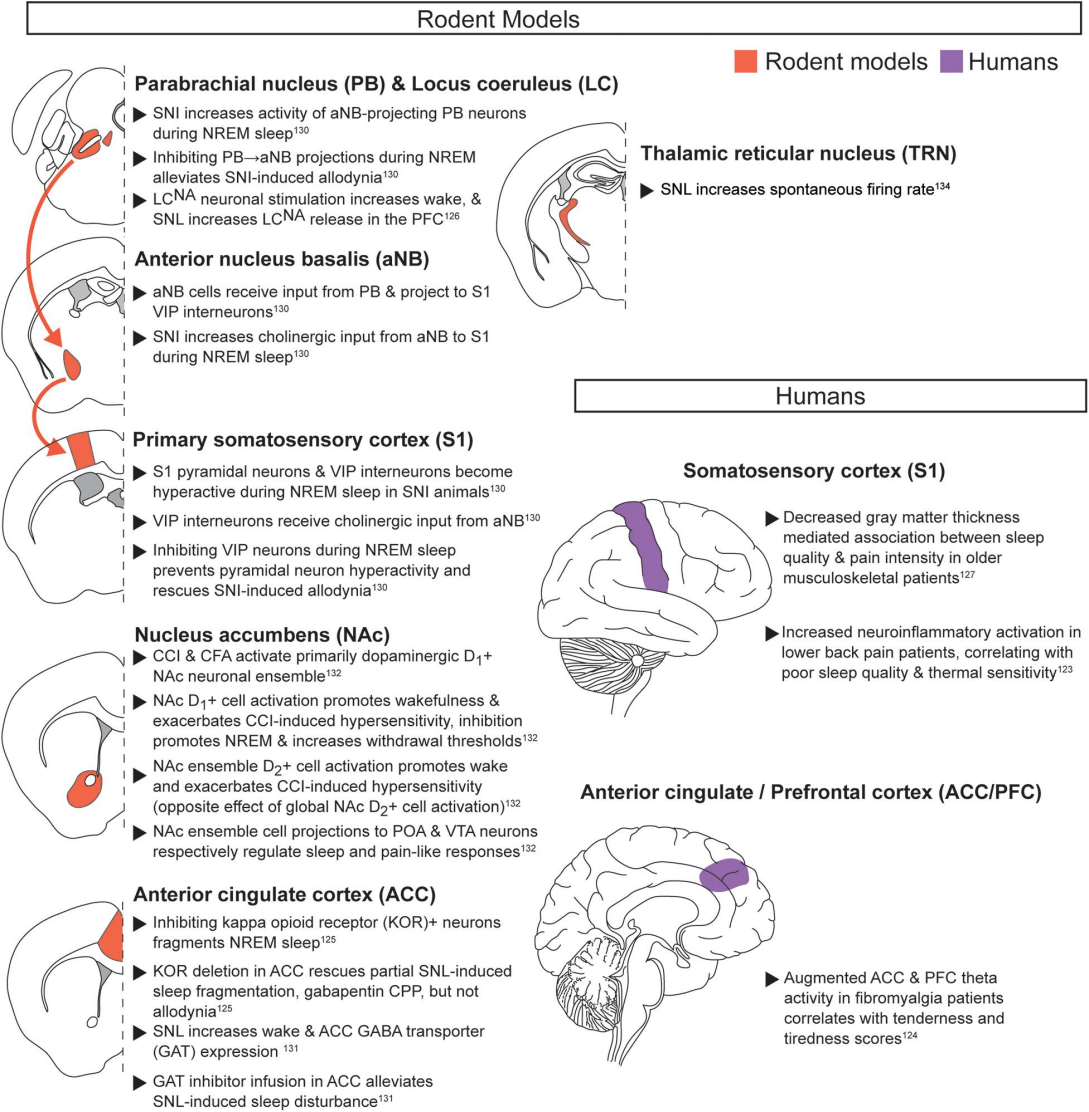
**Chronic pain during baseline sleep/wake.** Neuroanatomical regions implicated in regulating the interaction between pain and sleep in rodents and humans from studies evaluating chronic pain during baseline sleep and wake. Detailed descriptions are in the corresponding text section, and references are in superscript. SNI, spared nerve injury; VIP, vasoactive intestinal peptide-expressing; CCI, chronic constriction injury; CFA, Complete Freund's adjuvant; POA, preoptic area; VTA, ventral tegmental area; KOR, kappa opioid receptor; SNL, sciatic nerve ligation; CPP, conditioned place preference.

Chronic pain during baseline sleep/wake (Figure 4): While chronic pain conditions are highly varied in their symptoms and co-morbidities, preclinical and clinical studies highlight several brain regions that may regulate the consistent negative impact they have on sleep (123–134). As with acute noxious stimuli, the cortex has been implicated as a key region in chronic pain, with changes in long-term activity and structure that may influence sleep quality. In humans with chronic lower back pain, neuroinflammatory PET-fMRI activation was greater in lower back sensorimotor cortical areas (S1/M1), and was positively correlated with sensitivity to thermal stimuli and poor sleep quality (123). Changes in gray matter volume have also been shown. Older adults (>60 years old) with musculoskeletal pain exhibited decreased cortical thickness in S1, which mediated the association between sleep quality and self-reported pain intensity, but not somatosensory pain thresholds (127). Preclinical work has revealed possible sleep state-specific circuit mechanisms for chronic pain-induced changes in S1. In mice with spared nerve injury (SNI), S1 vasoactive intestinal polypeptide-expressing interneurons (S1^VIP^) were more active during NREM, leading to S1 pyramidal neuron disinhibition and allodynia (130). This study then rigorously described a circuit that can drive or alleviate SNI-induced pain-related behaviors with NREM specificity. More specifically, injured peripheral afferents increased parabrachial nucleus (PB) activity. PB neurons projected to basal forebrain cholinergic neurons (aNB^Ach^), which directly activated S1^VIP^ interneurons. Strikingly, hyperactivity of this pathway specifically during NREM sleep, not wake, promoted chronic SNI hypersensitivity. After SNI, daily inhibition of S1^VIP^ interneurons during NREM (but not during wake or REM) prevented the transition from acute to chronic SNI-induced pain-related behaviors (130). This PB → aNB^Ach^ → S1^VIP^ circuit offers a possible mechanism linking injured peripheral tissue to the hyperactive S1 commonly observed in chronic pain manipulations. However, further research must elucidate how this circuit interacts with spinally mediated peripheral neuropathic signaling, and how it regulates other chronic pain models.

Additional cortical regions besides S1 likely contribute to sleep and chronic pain interactions. In fibromyalgia patients, both the prefrontal cortex (PFC) and ACC show augmented theta activity that correlates with measures of somatosensory tenderness and mean tiredness (124). Preclinical work has revealed specific signaling pathways that can regulate activity in either of these regions. Increased PFC activity may be due to increased noradrenaline and serotonin signaling. In male mice with sciatic nerve ligation (SNL), stimulation of the LC or the DRN respectively increased noradrenaline or serotonin release in the PFC compared to non-SNL controls (126, 133). Together, these studies suggest that key sleep regulatory regions can potentially modulate PFC during chronic pain. Augmented ACC activity may be linked to opioid signaling. CRISPR/Cas9 deletion of kappa opioid receptors in the ACC alleviated partial sciatic nerve ligation (PSNL)-induced sleep fragmentation, as well as the increased place preference for gabapentin (125). However, PSNL-induced allodynia was unaffected, suggesting that somatosensory processing remains intact.

Altered inhibitory neurotransmission in cortex may also underlie impaired sleep during chronic pain. In male SNL mice with decreased NREM sleep, the cingulate cortex exhibited increased membrane-bound GABA transporters (GATs) and reduced extracellular GABA following depolarization (131). Furthermore, targeted GAT inhibition in the cingulate cortex attenuated SNL-induced sleep disturbance. However, the impact on pain-related behaviors remains to be evaluated (131).

The NAc has also been investigated in the context of baseline sleep behaviors during chronic neuropathic or inflammatory pain in mice. Dopaminergic neurons play an important role, as seen with acute noxious stimuli. A primarily dopaminergic NAc neuronal ensemble showed increased activity following chronic neuropathic pain, inflammatory pain induced by complete Freund's adjuvant, or during baseline wakefulness (132). Activating or inhibiting this ensemble respectively exacerbated or alleviated reflexive pain-related behaviors and sleep impairments, although these manipulations also promoted immediate transitions to wake or sleep. Interestingly, this NAc ensemble regulated pain and sleep through different target regions. Specifically silencing VTA-projecting neurons increased pain-related reflexive behaviors without impacting baseline wake behavior, while silencing preoptic area (POA)-projecting neurons decreased NREM sleep without impacting pain-related behaviors. Additional work must clarify how chronic pain regulates NAc ensemble activity, and whether projection-specific pain regulation applies to other pain modalities. However, this work provides an exciting entry point to understand key intersections in pain and sleep circuitry.

Finally, one study has shown that the reticular thalamic nucleus (RTN), a key regulator of NREM sleep oscillations, exhibits increased activity following neuropathic injury (134). Rats with SNL injury exhibited highly fragmented sleep and decreased total NREM time. In these animals, RTN basal tonic firing rates and phasic activity were both increased. However, additional research is needed to determine how these changes in RTN activity directly influence pain and sleep.

**Figure 5:**
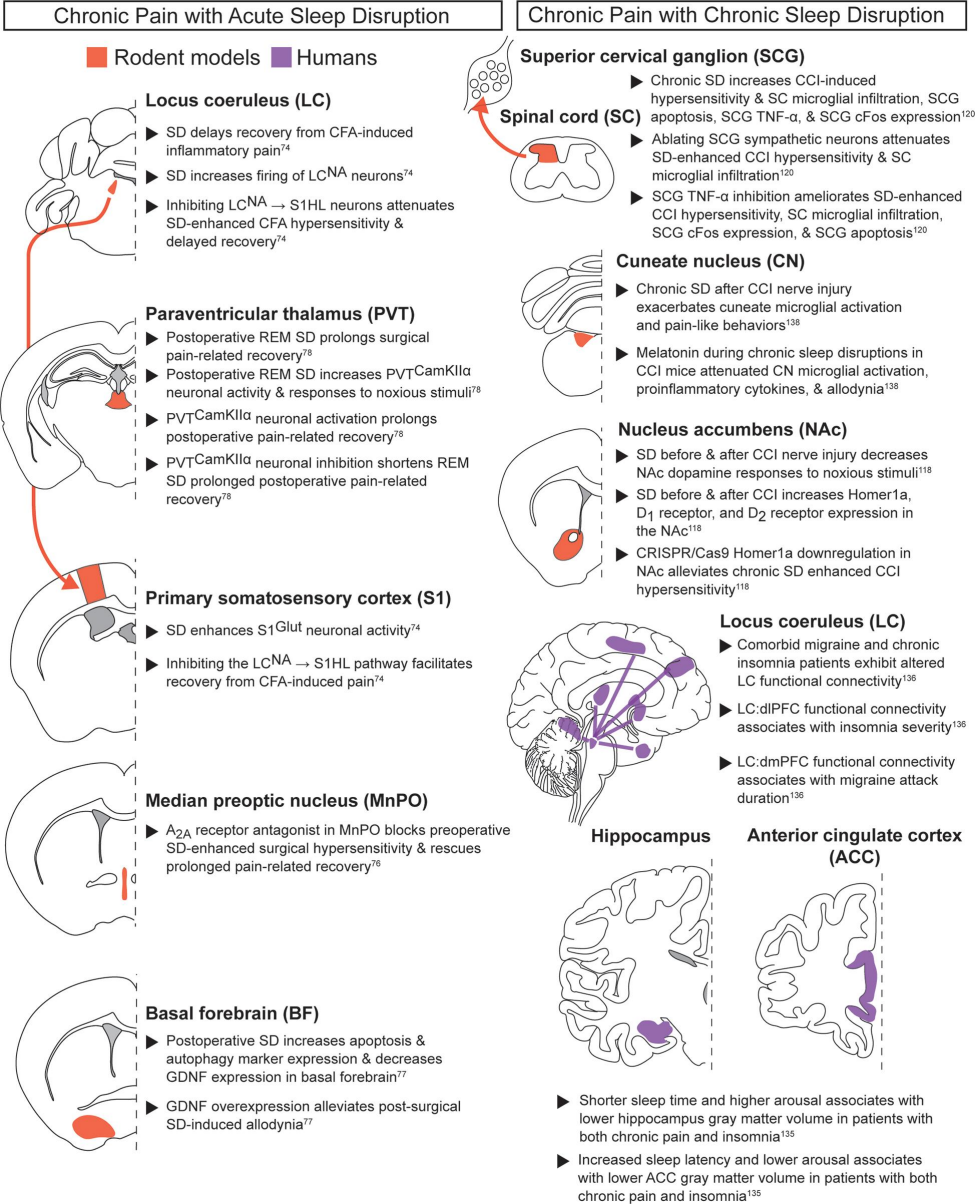
Chronic pain with acute or chronic sleep disruption. Neuroanatomical regions implicated in regulating the interaction between pain and sleep in rodents and humans from studies evaluating chronic pain paired with acute sleep disruption (left column) or chronic sleep disruption (right column). Detailed descriptions are in the corresponding text sections, and references are in superscript. SD, sleep disruption; CCK2, cholecystokinin 2; REM SD, REM-specific sleep disruption; NA, noradrenergic; CFA, Complete Freund's adjuvant; S1HL, hindlimb primary somatosensory cortex.

Chronic pain with acute sleep disruption (Figure 5): While clinical work has evaluated the relationship between chronic pain and acute sleep disruption, identification of underlying neuroanatomical mechanisms is based primarily on preclinical data. In agreement with other pain and sleep interactions, heightened activity within subcortical and cortical pathways may drive interactions between acute sleep disruptions and chronic pain. The hyperactivated LC^NA^ → S1HL^Glut^ pathway described above (see acute noxious stimuli with acute sleep deprivation) may also mediate hypersensitivity in the context of chronic inflammation (CFA) (74). Acute sleep deprivation five days after hindpaw CFA injection extended resulting hypersensitivity to at least 14 days post CFA injection, whereas control animals recovered in ∼6 days. Chemogenetically inhibiting LC^NA^ → S1HL neurons during the post-CFA sleep deprivation period attenuated the sleep deprivation-enhanced CFA hypersensitivity, thus shortening recovery time. Taken together, inhibiting the LC^NA^ → S1HL pathway may alleviate the effects of acute sleep deprivation on acute noxious stimuli or chronic inflammatory pain. These experiments thus pinpoint key interactions between known sleep and pain regulatory regions and also provide insights into the transition from acute to chronic pain.

The MnPO is another key sleep regulatory region that may play a role in transitioning to chronic pain following acute sleep deprivation. Sleep deprivation prior to surgery has been associated with increased post-surgical pain and delayed recovery, and clinical work has suggested perioperative sleep as an important metric for monitoring chronic pain susceptibility (8). Preclinical work suggests adenosine A_2A_ receptors in the MnPO may partially regulate the interactions between preoperative sleep deprivation and post-surgical pain. In rats, systemically administering caffeine or blocking MnPO adenosine A_2A_ receptors during preoperative sleep deprivation both alleviated sleep disruption-enhanced surgical hypersensitivity and improved postoperative pain-related recovery (76). Postoperative sleep disruption also augmented reflexive pain-related behaviors, prolonged pain-related surgical recovery, and altered the basal forebrain (BF), a key sleep-wake regulatory region (77). Post-surgical sleep disruption reduced GDNF expression and increased apoptosis in the BF. Virally delivering GDNF to the BF alleviated the effects of postoperative sleep deprivation, with reduced surgical recovery time, allodynia, and BF apoptosis. Thus, postoperative sleep deprivation may prolong hypersensitivity by decreasing GDNF signaling in the BF.

Prolongation of surgical pain due to perioperative sleep disruption has also been studied in REM-specific paradigms. Postoperative REM-specific sleep deprivation in mice prolonged surgical pain-related recovery and increased the activity of CaMKIIα neurons in the medial paraventricular thalamus (mPVT^CaMKIIα^) (78). These neurons were both necessary and sufficient for the prolonged post-surgical recovery after REM deprivation: mPVT^CaMKIIα^ neuronal inhibition shorted post-operative pain-related recovery, while activation prolonged recovery and produced anxiety-like behaviors. Cumulatively, these data highlight how perioperative sleep disruption can exacerbate pain-related recovery. Future work should determine how descending pain-modulatory regions (see section on acute noxious stimuli and acute sleep disruption section) regulate the interactions between perioperative sleep disruption and chronic pain.

Chronic pain with chronic sleep disruption (Figure 5): A large patient population suffers from comorbid chronic pain and chronic sleep impairment. Accordingly, a mechanistic understanding of chronic sleep and pain interactions is sorely needed (2, 6–9, 65). Consistent with previous pain and sleep subclasses, clinical studies suggest the cortex may play an important role in this bidirectional relationship. In adults with comorbid chronic pain and insomnia, individuals with the lowest arousal levels and the longest sleep onset latencies exhibited reduced ACC volume compared to healthy controls (135). This study also found that patients with shorter sleep times and higher arousal levels were associated with lower hippocampus volumes. These results suggest that distinct arousal and sleep parameters may differentially affect ACC and hippocampal structure in chronic pain patients.

Cortical alterations during comorbid chronic pain and chronic sleep disruption have been linked to the LC, a well-characterized regulator of sleep-wake states and a region repeatedly implicated across multiple pain and sleep subclasses. Neuroimaging studies in patients with comorbid migraine and insomnia revealed altered LC functional connectivity with PFC, with distinct PFC subdivisions showing specific pain and sleep associations (136). Lower LC → dorsolateral PFC functional connectivity of was associated with greater insomnia severity, while higher LC → dorsomedial PFC functional connectivity was linked to longer migraine attack duration, but only in migraine patients without insomnia. Based on the preclinical data linking LC to the PFC (see the chronic pain during baseline sleep/wake section), future studies should examine how chronic sleep deprivation modulates this NA signaling in different chronic pain models.

Beyond these cortical changes, subcortical areas, particularly the NAc, may play an important role in modulating concurrent chronic pain and chronic sleep deprivation. NAc dopaminergic neurons regulate sleep and pain through distinct projections (see chronic pain during baseline sleep/wake section). Recent preclinical work has extended these findings by testing how the NAc changes following chronic sleep disruption in a neuropathic pain model of chronic constriction injury (CCI) (118). Mice with CCI exhibited numerous changes in the NAc after 14 days of 18 h/day sleep disruption, including decreased dopamine release upon noxious stimulus application, increased Homer1a expression, increased D_1_ receptor expression, and increased D_2_ receptor expression. Downregulating Homer1a in the NAc alleviated the chronic sleep disruption-enhanced CCI hypersensitivity. Taken together, this suggests increased NAc Homer1a expression thus regulates both acute and chronic pain during extended sleep disruption. Collectively, these findings highlight the NAc as an important node between chronic pain and chronic sleep disruption. Future work can further dissect the relevant circuit mechanisms and their therapeutic relevance to other pain modalities.

Alongside central brain mechanisms, population-based and preclinical studies indicate that chronic sleep disruption can enhance chronic pain through spinal and peripheral inflammatory processes. A longitudinal prospective study monitoring >8,500 people for over ∼22 years (HUNT study) showed that individuals with short sleep or self-reported insomnia for >10 years were at higher risk of developing recurrent spinal pain, and improved sleep was associated with improved favorable prognosis (137). Chronic sleep disruption in CCI mice also affected the spinal cord, with greater microglial infiltration into the spinal cord dorsal horn than CCI alone, with concurrently increased apoptosis, TNF-α expression, and increased SCG cFos expression (120). As with acute noxious stimuli, inhibiting TNF-α in the SCG or ablating SCG sympathetic neurons attenuated chronic sleep deprivation-enhanced CCI mechanical hypersensitivity and microglial infiltration into the spinal cord. TNF-α inhibition also ameliorated chronic sleep deprivation increases in CCI-induced SCG cFos expression and apoptosis. Together, these findings show that TNF-α inhibition in the SCG is effective at alleviating impacts from chronic sleep disruption for both acute noxious stimuli and chronic neuropathic pain.

In addition to the spinal cord, brainstem sensory regions also appear to be vulnerable to microglial changes in chronic pain subjects with chronic sleep disruption. In particular, the cuneate nucleus (CN), which processes somatosensory input from the periphery, shows marked microglial activation in response to sleep deprivation. In male rats, chronic sleep disruption prior to CCI surgery led to greater microglial activation in the CN compared to CCI alone (138). Administering melatonin during chronic sleep deprivation attenuated both the CN microglial activation and allodynia, and also decreased proinflammatory cytokines expression in the CN. However, the impact of these melatonin doses to sleep behavior and behavioral depression remain to be tested.

In summary, pain and sleep interactions do not converge on the same circuits, but instead broadly engage multiple signaling pathways. While certain hubs such as S1, LC, and NAc contribute in multiple pain contexts, major gaps remain in our understanding of how these regions integrate information from cross-modal circuitry, and how their functions differ between the subclasses of pain and sleep interactions.

## Additional covariate considerations for treatment and biomarker development

Despite ongoing efforts to characterize neural circuits that mediate pain and sleep interactions, translation to the clinic for treating this bidirectional relationship remains limited. Including sleep in preclinical pain research provides a particularly promising opportunity for development of biomarkers and analgesic treatments. Primary sleep endpoints can be measured objectively and reproducibly across preclinical and clinical subjects. They also show well-established preclinical-to-clinical physiological concordance, and recent clinical work suggests they may be more effective for both treatment and prediction of chronic pain. Realizing this potential requires carefully designed studies that account for key covariates, which are abundant given the broad physiological impacts of pain and sleep. Accordingly, preclinical work can guide clinical studies by meticulously isolating variables and pinpointing key interdependencies. Below, we detail burgeoning work on variables such as sex, age, environmental context, stress, drug interactions, and choice of dependent measures, each of which can each profoundly impact pain and sleep outcomes. Accounting for these factors is therefore essential both for mechanistic insight and for improving the translational potential of preclinical models.

Sex differences: Responses to noxious stimuli differ between sexes, and mechanisms for processing peripheral nerve damage show striking differences (99, 139–141). Clinically, women show greater susceptibility to sleep disturbance and increased risk for developing pain conditions (142, 143). Additionally, prolonged experimental sleep disturbances may differentially affect pain processing in men and women (144, 145). Preclinical research has increasingly included female subjects, largely due to an NIH mandate for inclusion of sex as a biological variable. However, studies aiming to directly link pain and sleep have predominantly evaluated only male mice, as evidenced in the neural circuitry section above (146, 147). Accordingly, integrating sex as a core variable in preclinical studies will be crucial to refining mechanistic models of pain and sleep interactions and strengthening the translational validity of emerging biomarkers.

Age: Both pain and sleep show considerable changes across lifespan (148–150), and their interactions manifest at all ages with likely distinct mechanisms and health implications (151–159). In children and adolescents, sleep disruption and fatigue can predict next-day pain scores in subjects with pain and for healthy controls, mediate increased pain and sleep disturbances, and connect to multiple pain indications (152–154, 157). In addition, adolescent chronotype may predict future development of pain, with later chronotypes showing a higher risk for new-onset pain than earlier chronotypes (155). Older adult and elderly populations also show a clear link between pain and sleep disruption. Sleep quality and pain scores can predict care dependency in long-term care facilities, and sleep difficulty can mediate the relationship of daily activities and pain scores in middle-aged and older adults (156, 159). Data from adult populations suggest that insomnia treatment may be more efficacious for treating co-morbid pain and sleep disruptions that treating pain alone. However, further work must evaluate whether these findings also apply to young and elderly populations. Collectively, these findings highlight age as an important factor that should be included when establishing optimal strategies for reducing co-morbid pain and sleep disruptions.

Environment—Social Interaction: Social context can profoundly influence pain and sleep. Both preclinical and clinical evidence have shown noxious stimuli applied to one subject can elicit pain-related changes in the observer, also referred to as “emotional contagion” of pain (160–164). This includes increased pain responses, avoidance of behaviors paired with delivery of a noxious stimulus to a second subject, and increases in stress-response hormones that can even match those of the stressed subject (160, 162–164). Social familiarity can modulate these responses, as observation of pain in strangers elicits reduced or no empathetic physiological or behavioral pain-related changes. Similarly, social context can impact sleep behavior and sleep quality in humans and rodents. Positive social relationships strongly correlate with good sleep quality, while aversive social ties reliably predict poor sleep quality (164). For most mammals, sharing sleeping spaces contain intricate trade-offs. Group-living and shared sleeping environment produces behavioral synchronization in humans, rodents, and primates. However, it can also increase NREM fragmentation, decrease total NREM, or change REM and NREM bout length (33, 165–169). Specific shared sleeping environments like hospitals and military barracks are also associated with poorer sleep quality and additional adverse outcomes such as impaired recovery and wound healing (hospitals) or decreased quality of life (barracks) when compared to private rooms (167–172). Careful consideration of social environment in both study design and retrospective data analyses will be needed to better understand pain-sleep interactions and to develop effective clinical biomarkers.

Environment—Stress: Stress can independently influence either pain or sleep, and accumulating evidence suggests it may also mediate their bidirectional relationship (173–175). Clinically, sleep difficulties and stress can predict future chronic pain, and when combined confer heightened risk in adults and children (173, 174). Experimentally, stress is often divided into acute and chronic subcategories. Acute noxious stimuli commonly trigger acute stress, and acute stressors can in turn modulate responses to acute noxious stimuli (176–178). Both acute and chronic stressors can reliably exacerbate pain-related outcomes in subjects with a wide range of chronic pain conditions (176–178). Preoperative stress, like preoperative sleep, has been shown to predict development of postoperative chronic pain (6, 17, 18, 179). Stress can further impair sleep quality across species, with acute and chronic stressors reducing sleep both preclinically and clinically (70, 180, 181). While most sleep deprivation paradigms are not designed to induce stress, some have been shown to increase stress responses (70, 73). Given that sleep disruption and stress are inherently intertwined, particularly with prolonged or repeated sleep loss, disentangling their individual contributions remains a significant challenge. Preclinical studies will be critical to systematically isolate the effects of stress on pain-sleep interactions and guide the development of targeted clinical treatments.

Drug-drug interactions: Pharmacological interactions represent another covariate layer that can significantly influence pain and sleep outcomes. Analgesics such as opioids have been shown to impact sleep in undisturbed control settings, and drug-induced sleep disruptions can be detrimental for optimal sleep restoration, diminish pain-relief, and impair improvements in additional clinical outcome measures (53, 182). Accordingly, co-administration of drugs designed to individually treat either pain or sleep should be evaluated for unintended interactions, as treatment of one symptom can exacerbate the other (e.g., an analgesic with sedative effects further impairing sleep quality). Advancing research on the intersecting mechanisms underlying pain and sleep interactions will be essential in shaping pharmacological development, with the goal of enabling targeted therapeutic strategies and reducing reliance on complex polypharmacy approaches with mixed adverse effects (9, 21, 53, 67).

Interpreting preclinical dependent variables in pain and sleep: The clinical gold-standard for sleep evaluation is polysomnography, which measures EEG, EMG, eye movements (EOG), heart rhythm (ECG), pulse oximetry, airflow, and respiratory effort, which can all be measured preclinically (183). In contrast, the clinical gold-standard dependent variable for measuring pain is verbal reporting, which cannot be modeled in preclinical setups. Reflex-based assays of pain-related behaviors remain amongst the most widely used dependent variables in preclinical pain research, particularly for chronic pain manipulations. However, these assays are highly susceptible to false positives due to general behavioral depression. To improve clinical translation, extensive efforts have been made to supplement traditional reflexive pain-stimulated behaviors with spontaneous pain-related behaviors and pain-depressed behaviors in animal models (160, 184–186). In this context, leveraging directly translatable and well-characterized EEG and EMG analyses offer a uniquely promising preclinical-to-clinical avenue to identify pain-related biomarkers, with recent work in preclinical chronic pain models supporting their biomarker potential (69, 93). Pharmacological agents can affect normal sleep as an unintended side effect, with many classes of analgesics producing altered sleep architecture in the absence of any perturbations (21, 53, 67). Accordingly, restoration of normal sleep should be incorporated as a goal in preclinical analgesic testing. This will undoubtedly be more difficult than focusing solely on increasing or decreasing individual pain-related behaviors such as paw withdrawal, but may help focus research paths to more impactful pharmaceutical development. In addition, pharmacological investigations should evaluate changes to the spectral properties of sleep, particularly given ongoing clinical efforts evaluating distinct EEG frequency bands as candidate biomarkers for chronic pain (83–88). Together, these direct and translatable measures provide a framework in which efficacy of candidate analgesics will not only depend upon successful alleviation of pain-related behaviors but also on restoration of normal sleep architecture and spectral features.

Treatment goals: Clinical treatment goals for pain in relationship to sleep are primarily to improve quality of life, and future preclinical and clinical research will be essential to identify which approaches are most effective across different pain and sleep disorders. For conditions with known pain mechanisms, sleep provides a possible treatment for preventing chronification of acute pain. For example, both preclinical and clinical studies have shown that evaluating sleep before and after surgery can improve postoperative pain, recovery rate, and analgesic consumption (76–81, 187–189). For nociplastic pain conditions—defined as pain that arises despite no clear evidence of tissue injury or damage to the somatosensory system—sleep disturbances are a common co-morbidity (6, 65, 123, 190–192). Recent evidence from clinical studies indicates that treating insomnia with cognitive behavioral therapy for insomnia (CBT-I) can improve nociplastic pain outcome measures, and in some cases even out-perform CBT for pain (CBT-P) or combined CBT for insomnia and pain (CBT-IP) (6, 193–195). However, additional studies across diverse pain and sleep disorders are needed to confirm the broad implications of these findings and to evaluate the potential of insomnia treatment as a primary therapeutic strategy for comorbid chronic pain and sleep. Collectively, current pain and sleep studies underscore the importance of developing treatment goals that explicitly target both pain and sleep outcomes to maximize clinical benefit.

Summary: Positioning sleep as a core component of preclinical pain research holds promising potential for identifying robust biomarkers and therapeutic strategies with strong clinical translation. Advancing pain research through the lens of sleep will provide a preclinical roadmap to break the exacerbating cycles linking pain and sleep disturbances, and ultimately improve quality of life for patients worldwide.
